# Risk factors for Drug‐resistant Epilepsy (DRE) and a nomogram model to predict DRE development in post‐traumatic epilepsy patients

**DOI:** 10.1111/cns.13897

**Published:** 2022-07-12

**Authors:** Tingting Yu, Xiao Liu, Lei Sun, Ruijuan Lv, Jianping Wu, Qun Wang

**Affiliations:** ^1^ Department of Neurology, Beijing Tiantan Hospital Capital Medical University Beijing China; ^2^ China National Clinical Research Center for Neurological Diseases Beijing China; ^3^ Advanced Innovation Center for Human Brain Protection Capital Medical University Beijing China; ^4^ Collaborative Innovation Center for Brain Disorders, Beijing Institute of Brain Disorders Capital Medical University Beijing China

**Keywords:** drug‐resistant epilepsy, nomogram, post‐traumatic epilepsy, prognosis, risk factor

## Abstract

**Objectives:**

To identify factors affecting the development of drug‐resistant epilepsy (DRE), and establish a reliable nomogram to predict DRE development in post‐traumatic epilepsy (PTE) patients.

**Methods:**

This study conducted a retrospective clinical analysis in patients with PTE who visited the Epilepsy Center, Beijing Tiantan Hospital from January 2013 to December 2018. All participants were followed up for at least 3 years, and the development of DRE was assessed. Data from January 2013 to December 2017 were used as development dataset for model building. Those independent predictors of DRE were included in the final multivariable logistic regression, and a derived nomogram was built. Data from January 2018 to December 2018 were used as validation dataset for internal validation.

**Results:**

Complete clinical information was available for 2830 PTE patients (development dataset: 2023; validation dataset: 807), of which 21.06% (*n* = 596) developed DRE. Among all parameters of interest including gender, age at PTE, family history, severity of traumatic brain injury (TBI), single or multiple injuries, lesion location, post‐TBI treatments, acute seizures, PTE latency, seizure type, status epilepticus (SE), and electroencephalogram (EEG) findings, four predictors showed independent effect on DRE, they were age at PTE, seizure type, SE, and EEG findings. A model incorporating these four variables was created, and a nomogram to calculate the probability of DRE using the coefficients of the model was developed. The C‐index of the predictive model and the validation was 0.662 and 0.690, respectively. The goodness‐of‐fit test indicated good calibration for model development and validation (*p* = 0.272, 0.572).

**Conclusions:**

The proposed nomogram achieved significant potential for clinical utility in the prediction of DRE among PTE patients. The risk of DRE for individual PTE patients can be estimated by using this nomogram, and identified high‐risk patients might benefit from non‐pharmacological therapies at an early stage.

## Graphical abstract

In this article, 2830 patients diagnosed with post‐traumatic epilepsy (PTE) were reviewed and followed up. It was found that the risk factors for drug‐resistant epilepsy (DRE) included younger age at the first‐time late post‐traumatic seizure onset, focal onset or mixed onset seizure, the presence of SE, and epileptiform discharges during interictal EEG monitoring among PTE. A nomogram was constructed to effectively predict the risk of DRE for individual PTE patient. This is significant because the proposed nomogram achieved considerable potential for clinical utility in prediction of DRE among PTE patients. The risk of DRE for individual PTE patients can be estimated by using this nomogram, and identified high‐risk patients might benefit from non‐pharmacological therapies at early stage.

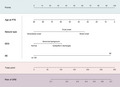

## 1 INTRODUCTION

Traumatic brain injury (TBI) is a global public health concern, with high morbidity, disability and fatality rate.^1^ Post‐traumatic seizure (PTS) and post‐traumatic epilepsy (PTE, defined as recurrent unprovoked PTS occurs more than 7 days after TBI) are common complications and sequelae after TBI.^2^ Early PTS also known as acute seizure, refers to seizures that occur within 7 days after TBI. Early intervention with antiseizure medications (ASMs) can effectively prevent acute seizure after TBI; however, it cannot prevent the development of PTE.^3^, ^4^ Most of the patients with PTE have a good prognosis, but there are still some patients who have poor responses to standard ASMs therapy and develop drug‐resistant epilepsy (DRE) with frequent seizures.^2^, ^5^ According to the numbers of published researches, the severity of TBI is a well‐established risk factor for PTE.^6^, ^7^, ^8^ Demographic factors (such as male gender, age ≥65 years),^9^, ^10^ specific TBI type and TBI characteristics (such as penetrating injury, skull fracture, hemorrhagic lesion, temporal lobe trauma, prolonged loss of consciousness, and post‐traumatic amnesia) ^2^, ^6^, ^9^, ^10^, ^11^ are also associated with the development of PTE after TBI. It is reported that PTE accounts for more than 5% of all patients with DRE transferred to epilepsy centers for surgical evaluation,^12^ and it takes a tremendous toll on patients both physically, mentally, and brings significant economic and mental burden to the family and society. Early prophylactic ASM therapy after TBI showed no protective effect on reducing the incidence of PTE.^13^, ^14^, ^15^ What is more, with the increase of ASM treatment options, the response of epilepsy patients to drugs gradually decreased,^16^ and the probability of developing DRE gradually increased. For PTE patients who are prone to develop into DRE and remain poorly controlled by early ASMs regimen, early comprehensive evaluation of epilepsy and alternative non‐pharmacological therapies (for example, vagal nerve stimulation [VNS], deep brain stimulation [DBS], or a potential resection operations) might help reduce the physical and mental damage of repeated seizures, so that the quality of life may improve. However, little attention has been paid to the prognosis of epilepsy patients who have developed PTE; there are few reliable large sample size studies to confirm the incidence and risk factors of DRE among PTE patients.^17^ It is imperative to identify risk factors for DRE and construct an accurate prognostic model to predict DRE development by longitudinally assessing a well‐powered sample of PTE patients.

A nomogram is a graphic score providing individualized predictions for clinical outcomes, such as the emergence of a disease or death.^18^ Nomograms are widely used for cancer prognosis,^18^, ^19^ and in recent years some researchers have also used them in clinical studies of neurological diseases.^20^, ^21^, ^22^ Till now, there is no nomogram for the prognosis of PTE that has been clearly characterized.

This study sought to identify the risk factors for DRE, and develop a nomogram model to predict DRE development in individual PTE patients.

## 2 METHODS

### 2.1 Study participants

This study retrospectively collected the medical records of patients diagnosed with PTE in the epilepsy center of Beijing Tiantan Hospital from January 2013 to December 2018. Inclusion criteria included: (1) definite TBI prior to seizure; (2) recurrent unprovoked PTS occurs more than 7 days after TBI, meet the diagnostic criteria of PTE; (3) complete medical record; (4) willing to follow‐up. Patients were excluded as follows: (1) perinatal injury, febrile convulsion, or seizure prior TBI; (2) pre‐existing neurological disease; (3) unclear medical record and cannot be remedied by follow‐up; (4) patients who had “undefined responsiveness” of ASMs.^23^

The study was approved by the Ethics Committee of the Beijing Tiantan Hospital affiliated with the Capital Medical University of the People's Republic of China. The study was conducted in accordance with the Declaration of Helsinki, and all participants provided informed consent for the use of their medical records.

### 2.2 Data collection

Data were collected through “the PTE patient information registration form” mentioned in our previously published study.^24^ In addition to demographic information, family history, personal medical records, TBI details, the clinical condition of PTE (including the presence of acute seizure, latency of PTE, the type and frequency of seizure), and the electroencephalogram (EEG), all patients were followed for at least 3 years. Close attention was paid to the usage of ASMs and the drug response of individual PTE patients. The response of ASMs was evaluated according to the seizure type and frequency before and after using appropriately chosen ASMs by two experienced neurologists (TTY and QW). Once the patient met the criteria of 2009 definition of DRE by ILAE: “failure of achieving a seizure‐free duration of 3 times the interseizure interval or 1 year (depending on which is longer) of two tolerated, appropriately chosen and used antiepileptic drugs (whether as monotherapies or in combination)”,^25^ he/she was assessed as developing DRE.

Concerning TBI, the severity was evaluated based on neurological and imaging evaluations.^6^ “Severe TBI” is characterized by one or more of the following features: brain contusion, intracranial hematoma, or loss of consciousness or post‐traumatic amnesia lasting ≥24 h. Otherwise, TBI was evaluated as “mild‐to‐moderate TBI”. This study also recorded the lesion location, and accessed the craniocerebral injury as a single injury or multiple injuries according to lesion caused by the TBI (single injury: a single or continuous lesion, for example, unilateral frontotemporal; multiple injuries: lesions of bilateral involvement or topographically separate locations, for example, bilateral frontal).^24^ Moreover, post‐TBI treatments were recorded, divided into conservative treatment, one surgical operation (puncture drainage or decompressive craniectomy during the acute phase of TBI), and multiple surgical operations (surgical operations during the acute phase plus another cranioplasty operation after the acute phase of TBI).

Latency of PTE referred to the time interval between TBI and the first‐time late PTS onset. Type of seizure was divided into generalized onset and focal onset seizure according to the 2017 classification of the International League Against Epilepsy (ILAE).^26^ The type of the most frequent seizures of each individual was recorded as his/her seizure type (generalized onset, focal onset, or mixed onset). According to the description of the patient or family members and the medical record, we evaluated whether the patient had status epilepticus (SE). The reference standard used was the 2015 classification of SE by ILAE.^27^ The type of seizure and the presence of SE was assessed solely based on the condition of the first 2 years of PTE course, the condition of 2 years later was not considered.

By reviewing the original EEG data or the report of EEG, this study recorded the EEG as “normal EEG”, “abnormal background without epileptiform discharges”, or “epileptiform discharges”. The EEG of all patients was any one of the interictal EEG during outpatient visit, and the duration of monitoring was 20–40 min.

Between September 2019 and August 2021, all patients were monitored in clinic or by telephone. All patients were followed continuously for at least 3 years before the last follow‐up. Modified Rankin Scale (mRS) score and the development of DRE were assessed at that time. Two neurologists (TTY and QW) participated in the assessment of the drug resistance status of the individual PTE patient.

### 2.3 Statistical analysis

SPSS 23.0 software (IBM Crop.) and R version 4.1.1 software were used for data analysis. Numerical data were represented by percentages, and continuous data were represented by mean ± standard deviation (SD) or median and interquartile range (IQR). The χ^2^ or Fisher exact test was used to compare numerical data as appropriate, and Mann–Whitney U test was used to compare continuous data. A two‐sided *p* < 0.05 was deemed significant. Univariate and multivariate logistic regression were used to identify factors have an impact on DRE.

### 2.4 Development and validation of Nomogram

The cohort of patients who visited our epilepsy center for the first‐time from January 2013 to December 2017 was used as the development dataset for model building; those from January 2018 to December 2018 were used as the validation dataset for internal validation. All candidate factors with *p* < 0.3 in univariate logistic regression analysis were included in the initial multivariable logistic regression. Then non‐significant predictors were eliminated in a stepwise fashion. The final model was the model corresponding to the minimum Akaike information criterion (AIC). The nomogram was developed using the final multivariable logistic regression analysis results, based on R version 4.1.1 software. In the nomogram model, the regression coefficient of each predictor was used to determine the proportion of scores; 100 points were assigned to the predictor with the highest regression coefficient, the other predictors were given corresponding points based on weight. For each patient, a total score was given by summing the scores received from different predictors. Each total score could be converted into the predicted risk of DRE. The higher the score, the greater the risk of developing DRE. Subsequently, the model was evaluated based on nomogram discrimination and calibration. Areas under the receiver operating characteristic curves (AUC) were calculated to form the C‐indexes for evaluating discrimination, and we drew calibration curves and visualized Hosmer‐Lemeshow goodness‐of‐fit test for evaluating calibration. *p‐*value >0.05 indicates good calibration.

## 3 RESULTS

### 3.1 Patient characteristics

**FIGURE 1:**
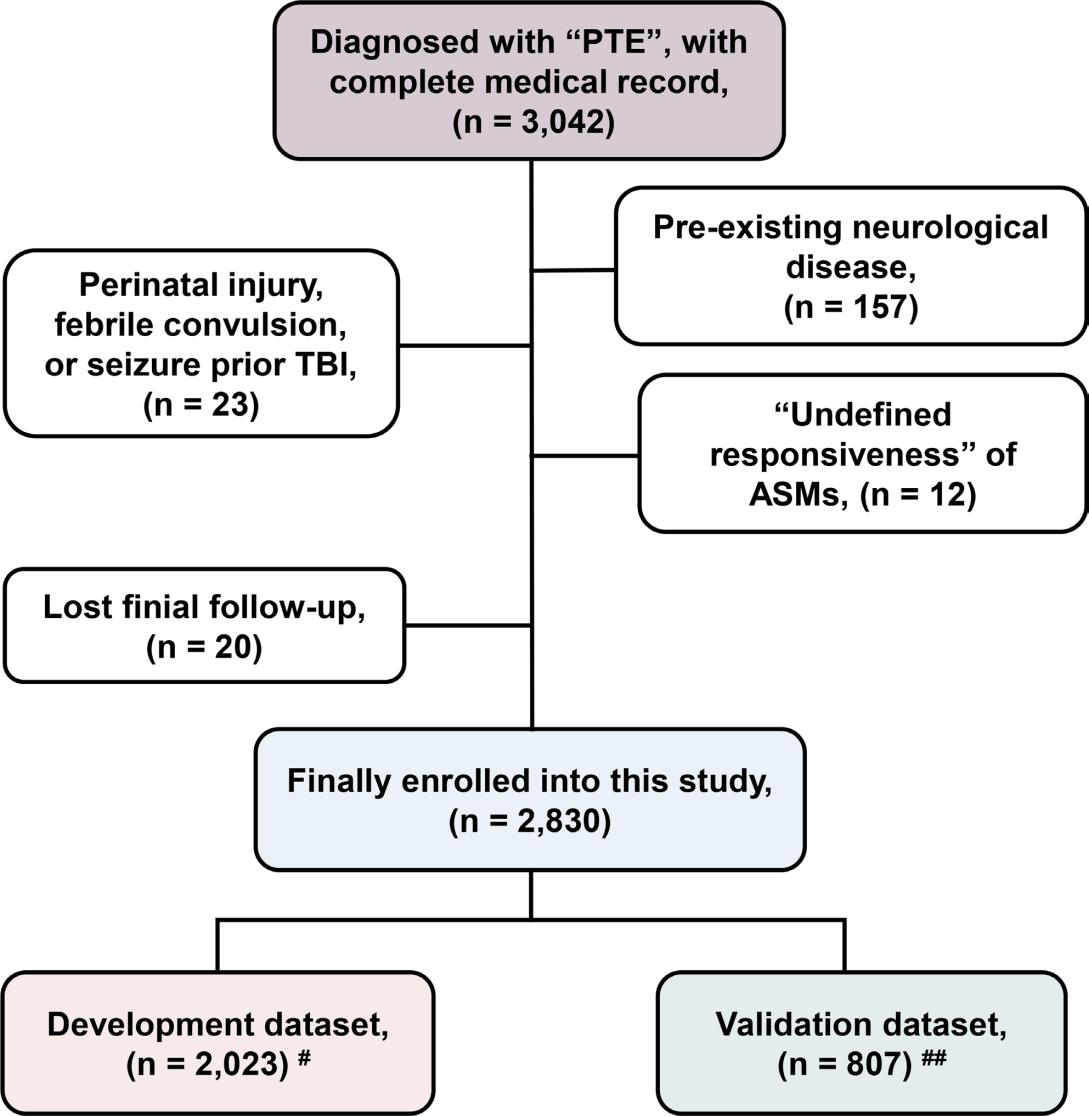
Case screening and datasets partitioning flow diagram. PTE, post‐traumatic epilepsy; TBI, traumatic brain injury, ASMs, antiseizure medications. ^#^Data from January 2013 to December 2017 were used as development dataset for model building; ^##^Data from January 2018 to December 2018 were used as validation dataset for internal validation

The clinical data of all patients diagnosed with “PTE” at our epilepsy center between January 2013 and December 2018 were reviewed, and 3042 patients who met the inclusion criteria mentioned above were screened. 212 patients were excluded because of the exclusion criteria mentioned above, data from 2830 patients with PTE were finally included in this study for analysis (Figure 1). The median age at TBI was 20.0 (IQR, 9.0–20.0) years. The latency ranged from 15 days to 20 years, with a median of 24.0 (IQR, 5.0–84.0) months. The median age of the first‐time late PTS onset (express as age at PTE) was 23.4 (IQR, 15.0–34.1) years, and the course of PTE was 9.0 (IQR, 6.0–16.0) years at the last follow‐up. Of all the patients enrolled in this study, 21.06% (596/2830) developed DRE. 11.02% (312/2830) had an mRS score of >2 and were assessed as having a disability. According to the rules mentioned above, 2023 of the 2830 patients were included in the development dataset; the other 807 patients were included in the validation dataset (Figure 1). Patients were divided into two groups (no‐DRE group: patients did not develop DRE; DRE group: patients had developed DRE) according to their DRE development. Table 1 shows the characteristics of the two groups within the development, validation, and entire datasets.

**TABLE 1:** Patients characteristics of no‐DRE group vs. DRE group

	Entire dataset (*n* = 2830)			Development dataset (*n* = 2023)			Validation dataset (*n* = 807)		
	no‐DRE (*n* = 2234)	DRE (*n* = 596)	*p*‐Value	no‐DRE (*n* = 1602)	DRE (*n* = 421)	*p*‐Value	no‐DRE (*n* = 632)	DRE (*n* = 175)	*p*‐Value
Demographics									
Gender (male)	1775 (79.5%)	447 (75.0%)	0.019*	1270 (79.3%)	317 (75.3%)	0.077	505 (79.9%)	130 (74.3%)	0.108
Age at PTE (yr)	24.0 (16.0–35.0)	21.0 (13.0–32.0)	0.000**	23.3 (16.0–33.0)	20.0 (13.0–30.0)	0.000**	26.0 (16.6–40.0)	24.8 (13.0–35.0)	0.008*
Course of PTE	9.0 (6.0–16.0)	9.0 (6.0–17.0)	0.848	10.0 (7.0–17.0)	10.0 (7.0–17.0)	0.405	6.0 (3.9–12.8)	6.0 (3.0–12.8)	0.500
Family history	13 (0.6%)	6 (1.0%)	0.261	11 (0.7%)	4 (1.0%)	0.531	2 (0.3%)	2 (1.1%)	0.168
TBI details									
Severe TBI	1182 (52.9%)	293 (49.2%)	0.104	827 (51.6%)	207 (49.2%)	0.370	355 (56.2%)	86 (49.1%)	0.098
Multiple injuries	1109 (49.6%)	310 (52.0)	0.304	778 (48.6%)	22 (52.7%)	0.128	331 (52.4%)	88 (50.3%)	0.625
Location									
Outside TL	863 (38.6%)	202 (33.9%)	0.018*	652 (40.7%)	157 (37.3%)	0.148	211 (33.4%)	45 (25.7%)	0.107
Left TL	621 (27.8%)	199 (33.4%)		363 (22.7%)	114 (27.1%)		258 (40.8%)	85 (48.6%)	
Right TL	750 (33.6%)	195 (32.7%)		587 (36.6%)	150 (35.6%)		163 (25.8%)	45 (25.7%)	
Post treatment									
Conservative	1246 (63.8%)	408 (68.5%)	0.109	1053 (65.7)	291 (69.1%)	0.286	373 (59.0%)	117 (66.9%)	0.059
Single operation	489 (21.9%)	115 (19.3%)		353 (22.0%)	78 (18.5%)		136 (21.5%)	37 (21.1%)	
Multiple operations	319 (14.3%)	73 (12.2%)		196 (12.2%)	52 (12.4%)		123 (19.5%)	21 (12.0)	
Acute seizure	127 (5.7%)	42 (7.0%)	0.212	88 (6.2%)	32 (7.6%)	0.291	28 (4.4%)	10 (5.7%)	0.478
PTE details									
Latency	24.0 (6.0–84.0)	24.0 (4.0–84.0)	0.993	24.0 (6.0–84.0)	24.0 (3.0–72.0)	0.249	24.0 (6.0–84.0)	9.0 (4.5–17.0)	0.078
Seizure type									
Generalized onset	1675 (75.0%)	349 (58.6%)	0.000**	1232 (76.9%)	258 (61.3%)	0.000**	443 (70.1%)	91 (52.0%)	0.000**
Focal onset	295 (13.2%)	112 (18.8%)		197 (12.3%)	72 (17.1%)		98 (15.5%)	40 (22.9%)	
Mixed onset	264 (11.8%)	135 (22.7%)		173 (10.8%)	91 (21.6%)		91 (14.4%)	44 (25.1%)	
SE	109 (4.9%)	110 (18.5%)	0.000**	82 (5.1%)	72 (17.1%)	0.000**	27 (4.3%)	38 (21.7%)	0.000**
EEG									
Normal	446 (20.0%)	82 (13.8%)	0.001*	302 (18.9%)	50 (11.9%)	0.001*	144 (22.8%)	32 (18.3%)	0.399
Abnormal background	287 (12.8%)	69 (11.6%)		197 (12.3%)	45 (10.7%)		90 (14.2%)	24 (13.7%)	
Epileptiform discharges	1501 (67.2%)	445 (74.7%)		1103 (68.9%)	326 (77.4%)		398 (63.0%)	119 (68.0%)	
mRS >2	252 (11.3%)	60 (10.1%)	0.401	179 (11.2%)	44 (10.5%)	0.674	73 (11.6%)	16 (9.1%)	0.368

*Note*: *p < 0.05; **p < 0.01.

Abbreviations: DRE, drug‐resistant epilepsy; EEG, electroencephalogram; LT, temporal lobe; mo, months; mRS, modified Rankin Scale; PTE, post‐traumatic epilepsy; RT, right temporal lobe; SE, status epilepticus; TBI, traumatic brain injury; yr, years.

As shown in Table 1, in the entire dataset, six variables showed significant differences between the no‐DRE group and the DRE group: gender (*p* = 0.019), age at PTE (*p* < 0.001), lesion location (*p* = 0.018), the type of seizure (*p* < 0.001), the presence of SE (*p* < 0.001), and the findings of EEG (*p* = 0.001). None of the following variables resulted in a significant difference between the two groups: family history, severity of TBI, single or multiple craniocerebral injuries, post‐TBI treatment, the presence of acute seizure, and latency of PTE. There was no difference in mRS score between the two groups.

### 3.2 Risk factors for DRE

In order to identify potential risk factors for DRE, we performed a univariate logistic regression for each variable in the development dataset (Table 2). Since the post‐TBI treatment was significantly correlated with the severity of TBI, we combined the two variables together for analysis, represented by the variable “severity‐treatment” (stratified into four subgroups: mild‐to‐moderate TBI, severe TBI with conservative treatment, severe TBI with one surgical operation, and severe TBI with multiple surgical operations). Variables with *p* < 0.3 included gender, age at PTE, lesion location, multiple injuries, severity‐treatment of TBI, the presence of acute seizure, type of seizure, the presence of SE, and EEG funding (Table 2). Those nine variables with *p* < 0.3 were entered into the initial multivariable logistic regression, after eliminating, four of them were remained in the final logistic regression model: age at PTE, type of seizure, the presence of SE, and EEG findings. All terms in the final model were statistically significantly related to the development of DRE (*p* < 0.05) (Table 3).

**TABLE 2:** Univariate logistic regression of DRE development

Variable	OR	OR 95% CI	*p*‐Value
Gender, female	1.255	0.972–1.611	0.077*
Age at PTE (year)	0.983	0.975–0.991	0.000**
Family history	1.387	0.383–4.080	0.577
Severity‐treatment			
Mild‐to‐moderate TBI	Ref		
Severe TBI with conservative treatment	1.003	0.744–1.342	0.984
Severe TBI with single surgery	0.800	0.597–1.063	0.129*
Severe TBI with multiple surgeries	0.961	0.678–1.342	0.818
Multiple injuries	1.182	0.953–1.465	0.128*
Injury location			
Outside temporal lobe	Ref		
Left temporal lobe	1.304	0.991–1.713	0.057*
Right temporal lobe	1.061	0.584–1.362	0.641
Acute seizure	1.248	0.814–1.868	0.292*
Latency (month)	0.999	0.997–1.001	0.397
Seizure type			
Generalized onset	Ref		
Focal onset	1.745	1.285–2.350	0.000**
Mixed onset	2.512	1.880–3.341	0.000**
Presence of SE	3.824	2.726–5.257	0.000**
EEG			
Normal	Ref		
Abnormal background	1.380	0.886–2.144	0.153*
Epileptiform discharges	1.785	1.302–2.491	0.000**

*Note*: *p < 0.30; **p < 0.01.

Abbreviations: CI, confidence intervals; DRE, drug‐resistant epilepsy; EEG, electroencephalogram; OR, odds ratio; PTE, post‐traumatic epilepsy; Ref, reference; SE, status epilepticus; TBI, traumatic brain injury.

**TABLE 3:** Final multivariable logistic regression of DRE development

Variable	*β*‐Coefficient	SE	OR (95% CI)	*p*‐Value
Intercept	−1.695	NA	NA	NA
Age at PTE (year)	−0.015	0.004	0.985(0.977–0.993)	0.000**
Seizure type				
Generalized onset	Ref			
Focal onset	0.551	0.158	1.736(1.267–2.358)	0.000**
Mixed onset	0.850	1.150	2.339(1.738–3.133)	0.000**
Presence of SE	1.289	0.176	3.628(2.577–5.120)	0.000**
EEG				
Normal	Ref			
Abnormal background	0.287	0.231	1.333(0.846–2.096)	0.213
Epileptiform discharges	0.469	0.170	1.598(1.155–2.249)	0.006**

*Note*:**p < 0.01.

Abbreviations: CI, confidence intervals; DRE, drug‐resistant epilepsy; EEG, electroencephalogram; NA, not applicable; OR, odds ratio; PTE, post‐traumatic epilepsy; Ref, reference; SE, standard error; SE, status epilepticus.

### 3.3 Nomogram model development and validation

**FIGURE 2:**
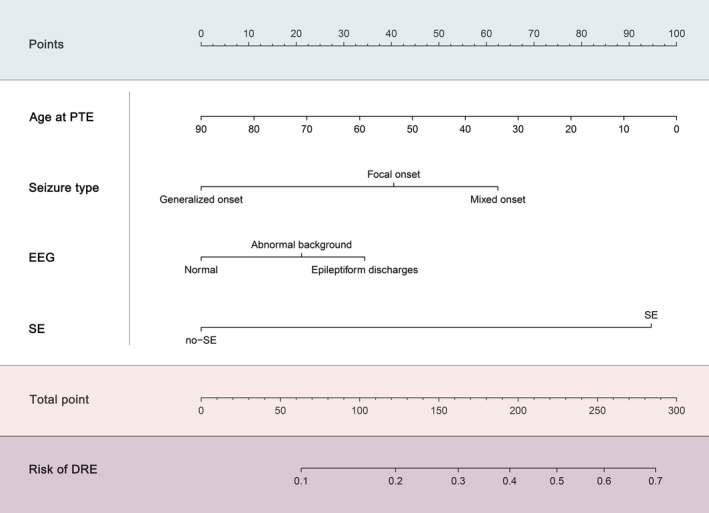
Nomogram for prediction of developing DRE risk among PTE patients. Determine individual risk in three steps: Step 1, for every variable on the left, count the points given at the top; Step 2, add up the points to a total score; Step 3, Determine associated risk of DRE. For example, a patient had the first‐time late PTS onset at 30 years old (66 points), had focal onset seizures (40 points), had a history of SE (95 points), and epileptiform discharges (35 points) were found on EEG, has a total of 236 points, which corresponds to a risk of developing DRE of 48%. DRE, drug‐resistant epilepsy; EEG, electroencephalogram; PTE, post‐traumatic epilepsy; PTS, post‐traumatic seizure; SE, status epilepticus

A model incorporating these four characteristics was created according to the multiple logistic regression results, and a nomogram to calculate the probability of DRE using the coefficients of the model was developed (Figure 2). In this nomogram, the predictor of age at PTE was assigned of 100 points, followed by the presence of SE, and the EEG findings had the least effect on DRE development. This nomogram provides convenience when predicting the probability of DRE. For individual PTE patients, we first identify the position of each variable on the corresponding axis, then we sum the points for each variable to form a total score by drawing lines to the points axis. The total score axis (ranged from 0 to 300 points) is used to estimate the probability (ranged from 10% to 70%) of DRE for each given patient.

**FIGURE 3:**
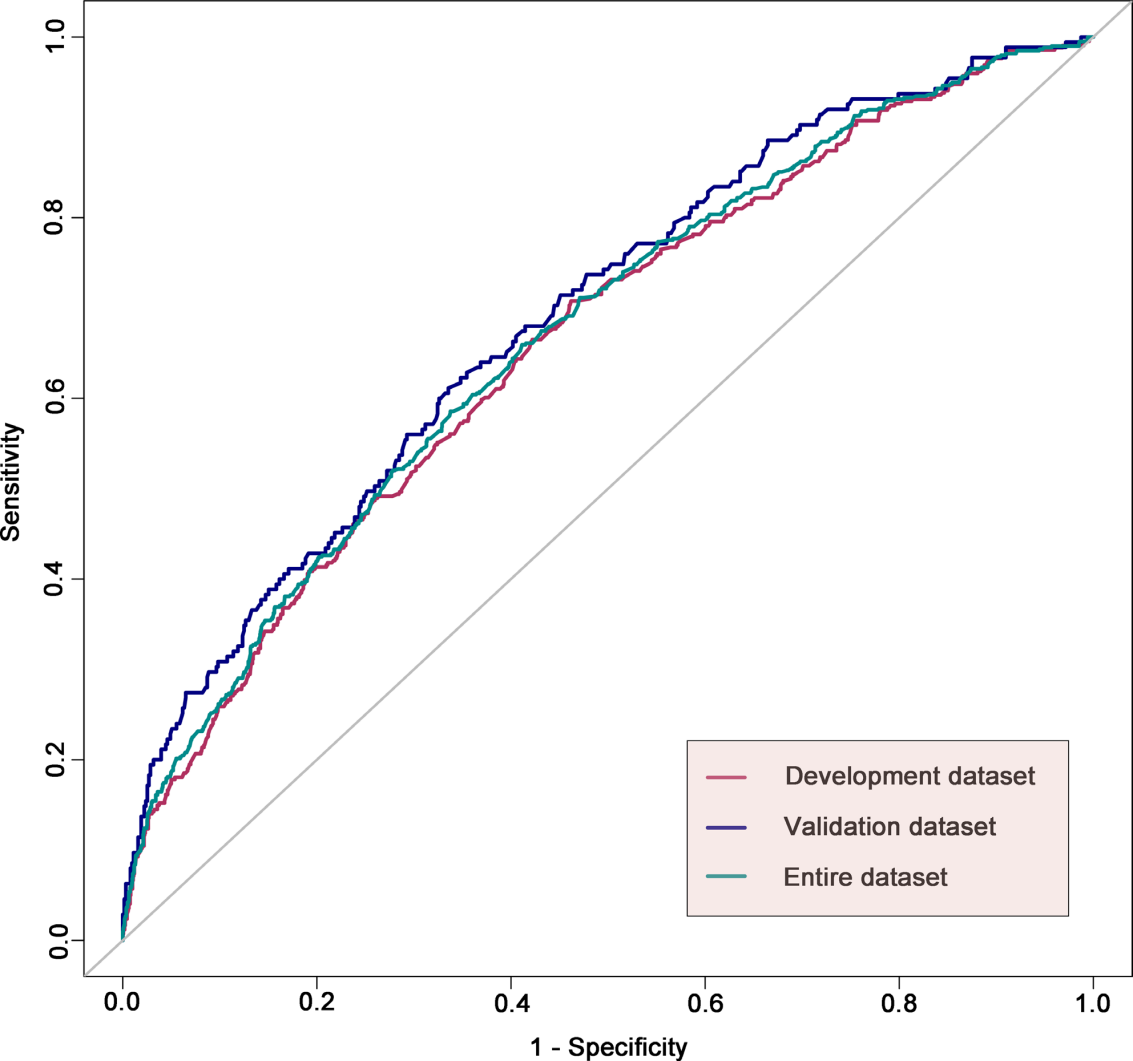
ROC curves of the nomograms. ROC curves of the nomograms in the development dataset, validation dataset and entire dataset. The nomogram had acceptable discriminative power with an AUC of 0.662 (95% CI: 0.633–0.691), 0.690 (95% CI: 0.645–0.734), 0.670 (95% CI: 0.646–0.695) in the development dataset, validation dataset and entire dataset, respectively. AUC, area under the receiver operating characteristic curve; ROC, receiver operating characteristic

**FIGURE 4:**
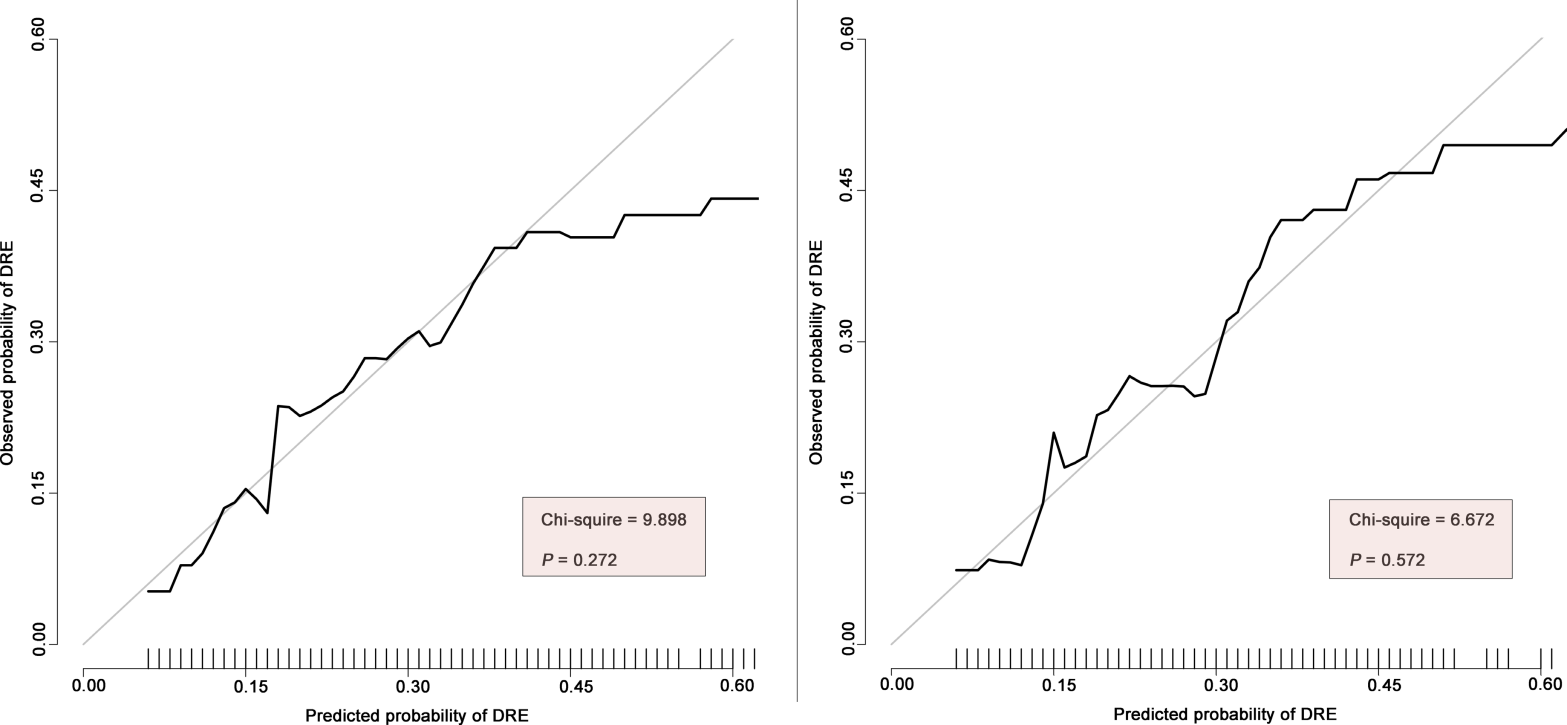
Calibration curves. Calibration curves of predicted probability of DRE (x‐axis) vs. observed probability (y‐axis), in development dataset (*p* = 0.272) (left), and validation dataset (*p* = 0.572) (right). The histogram at the bottom of the plot shows the distribution of the predicted. The 45° reference line indicates that the predicted probability is completely consistent with the observed probability. The Hosmer‐Lemeshow goodness‐of‐fit test was used to compare predicted probability and observed probability, *p‐*value > 0.05 indicates good calibration. DRE, drug‐resistant epilepsy

This nomogram demonstrated acceptable accuracy in estimating the risk of DRE among PTE patents, with a C‐index of 0.662 (95% CI, 0.633–0.691) for the development dataset, 0.690 (95% CI, 0.645–0.734) for the validation dataset, and 0.670 (95% CI, 0.646–0.695) for the entire dataset (Figure 3). The calibration curves for both the development and validation datasets exhibited a closed agreement between the predictions and the actual observations (Figure 4). The H‐L goodness‐of‐fit test χ^2^ statistic was 9.898 (*p* = 0.272) and 6.672 (*p* = 0.572) in the development and validation datasets, respectively, indicating good calibrations.

## 4 DISCUSSION

As one of the most common and severe complication of TBI, PTE leads to significant psychosocial and economic burden, psychiatric comorbidity, physical damage, low quality of life, and sudden unexpected death of the patients,^11^, ^28^ especially for those that are progressing to DRE.^29^, ^30^ In an era when everyone is paying more attention to the quality of life, patients and physicians would benefit greatly from the ability to predict the development of DRE at the early stage of PTE. However, there is a lack of investigations on the risk factors for DRE among PTE patients based on a large sample size, or a reliable model that helps to predict the risk of DRE for individual PTE patients. In the current study, 2830 patients diagnosed with PTE were reviewed and followed up. It was found that the independent risk factors for DRE included younger age at the first‐time of late PTS onset, focal onset or mixed onset seizure type, the presence of SE, and epileptiform discharges during interictal EEG monitoring. Using the set variables, a nomogram was constructed to predict the risk of DRE for individual PTE patients.

Over the years, there have been many published literatures,^16^, ^22^, ^31^ which have carried out in‐depth exploration on the incidence, risk factors, and outcomes of DRE. However, DRE caused by a specific etiology (such as TBI) may not have been explored. The incidence of DRE varies from 15% to 34% in patients with epilepsy,^32^ this study found a 21.06% incidence of DRE among PTE patients, which is consistent with the previous studies. A higher incidence of DRE also has been reported in patients diagnosed with symptomatic epilepsy with a clear etiology;^32^ however, the definitions of DRE in these studies were inconsistent. It is important to adopt homogenous criteria to assess the efficacy of ASMs in order to improve the generalizability of the study findings. Therefore, the 2009 definition of DRE by ILAE^25^ was used in this study. We realize that the course of epilepsy is critical for the assessment of the drug resistance status, as some epilepsy patients might have an excellent response to ASMs at the early stages of epileptic seizures, but would gradually appear ASMs resistant as the disease progresses.^33^ Observation duration is also an important link in the diagnosis and evaluation of DRE. DRE might be misdiagnosed in patients whose seizures are well controlled after two tolerated, and appropriately chosen ASMs therapies with insufficient observation duration. Ramos‐Lizana et al.^34^ followed 508 children with epilepsy for 14 years, and found the probability of DRE was 11%, 11%, and 13% at 2, 6, and 10 years, respectively. His research suggested that at least 2 years of observation might be appropriate from the perspective of epilepsy patients progressing to DRE,^34^ and 3 years might be more appropriate from the perspective that some patients may reach seizure remission again after diagnosis. Therefore, the follow‐up duration of this study was appropriate. Patients included in this study had a course of PTE ranging from 3 years to 66.8 years, with a median of 9.0 (6.0–16.0) years. Thus, the of the drug resistance status of individual PTE patients in this study was reliable.

For patients who have developed DRE, non‐pharmacological therapies are alternative options, which mainly include VNS, DBS, potential resection operations, and laser‐induced thermal therapy. ^35^ Numerous studies have reported that VNS effectively reduce seizure frequency in DRE patients as an adjunctive therapy,^36^ it was also reported that VNS can cause spontaneous neural activity changes in an ongoing process.^37^ DBS in the appropriate stimulation targets (such as medial septum for temporal lobe epilepsy) was also reported not only reduced spontaneous seizures, but also improved behavioral performance.^38^ These results suggest that if PTE patients at high risk of DRE could be identified and treated with non‐pharmacological therapies in the early stage, they might benefit early, and non‐pharmacological therapies in the early stage might help reduce the physical and mental damage of repeated seizures, improve their quality of life.^35^ This also underscores the importance of identifying patients at high risk for DRE.

The results of this study indicated that demographic characteristics played roles in predicting the progression of PTE patients to DRE. We found that female patients tended to be more likely to develop DRE than male patients (though the difference was not statistically significant). This was consistent with previous findings that females were at a higher risk for PTE after TBI.^7^ The effect of age on PTE or DRE is controversial. Christensen,^7^ Annegers,^6^ and Zhao^39^ reported that older age were risk factors for PTE, while younger age at seizure onset was reported as a risk factor for DRE.^32^ This study found that patients who had the first late PTS onset at a younger age had a higher risk of developing DRE.

According to a meta‐analysis published by Kalilani et al.^32^ risk factors for DRE included a history of febrile seizure (1.31, 95% CI 1.02–1.68), SE (3.30, 95% CI 2.36–4.63), abnormal EEG (2.08, 95% CI 1.16–3.74), and abnormal neuroimaging test results (2.78, 95% CI 1.91–4.05), while focal onset seizure was tensed to have a higher risk than generalized onset seizure (1.29, 95% CI 0.75–2.19). In another meta‐analysis published in recent years,^40^ the authors pointed out that abnormal EEG (both slow wave and epileptiform discharges) (2.80, 95% CI 1.95–4.00), SE (11.60, 95% CI 7.39–18.22), multiple seizure types (3.66, 95% CI 2.37–5.64), and febrile seizures (3.43, 95% CI 1.95–6.02) were risk factors for DRE. SE, abnormal EEG, focal, or multiple seizure types were reported playing important roles in DRE development in both of the studies.^32^, ^40^ In our cohort of PTE patients with TBI as the etiology, SE, abnormal EEG, focal onset seizures, or mixed onset seizures were also found to be risk factors for DRE, and those factors were eventually included in the nomogram model. Among those factors, the presence of SE had the greatest weight in predicting the development of DRE, which is consistent with the reported results.^32^ The type of seizure also had a significant predictive effect on DRE: patients who had mixed onset seizures were most likely to develop DRE, followed by focal onset seizure. EEG, has an established value for diagnosing and classifying epilepsy; however, it has an unclear role in predicting clinical outcomes.^41^ We found abnormal findings of interictal EEG monitoring were associated with DRE among PTE patients, especially the presence of epileptiform discharges. Previous studies also reported that focal epileptiform activity,^42^ focal slowing, asymmetric spike–wave discharges,^42^ and EEG with generalized epileptiform activity (primarily poly‐spikes) ^31^ were poor prognostic factors for epilepsy. Therefore, we believe that EEG monitoring, even routine interval EEG monitoring, is of great value in assessing the outcome of PTE.

This study found that in a cohort of patients with PTE, temporal lobe lesion location (especially left temporal lobe) tented to be related to a higher risk of DRE; however, the difference was not statistically significant in the final multivariable regression after correction. The temporal lobe, where PTE most frequently arises,^43^ lowers the seizure threshold and susceptibility.^44^, ^45^ Previous literature indicated that the underlying medial structures of the temporal lobe region were associated with DRE after TBI.^43^ Hunt et al.^46^ reported that injury to the underlying structures may disrupt normal neural processing, engendering the construction and maturation of an epileptogenic network over time. Hitti F et al.^47^ summarized 23 PTE patients who underwent surgical treatments for DRE, found that 82.6% had mesial temporal sclerosis. Thus, the temporal lobe might be an important area of focus for future studies assessing the genesis and progression of PTE.^11^ Interestingly, other features of TBI, which thought to be associated with the development of PTE after TBI, such as the severity of TBI,^6^ multiple injuries,^48^ and multiple surgical operations, were not associated with the development of DRE.

Among all patients enrolled in this study, 11.02% (312/2830) of them had a disability (mRS score >2), with no difference between the no‐DRE group and the DRE group. We considered the disability of the patients in this study a reflection of the severity of TBI. What is more, DRE and disability might be mutually reinforcing, but further investigation is required to confirm this hypothesis.

Our study has several strengths. First, the DRE of individual PTE patients was assessed strictly in accordance with the 2009 definition of DRE by ILAE,^25^ which improves the generalizability of the study findings. Second, based on a large sample size, our study identified the risk factors for the development of DRE among PTE patients, and also visualized the findings by developing a nomogram for the first time. The nomogram provided a more individualized prediction of the development of DRE for each PTE patient. Third, though the C‐index (0.65–0.70) was not very high on the absolute scale, the nomogram is based on age, easily ascertainable clinical and routine interictal EEG characteristics, making it a suitable prediction model as well as being easily integrated into daily clinical practice for PTE patients during the early stages of progression. Moreover, the model exhibited acceptable predictive capability in the development and validation datasets, which lends credibility to its usefulness.

Using a predictive tool helps identify PTE patients at a high risk of developing DRE, who might need closer and more regular monitoring. We physicians might make a more individualized, aggressive plan (for example, VNS, DBS, or resection operations) for those patients at the early stage of PTE.

As a retrospective study, we also realized that this study has several limitations. First, there might be some unobserved and/or uncontrolled confounding factors and we might miss a few factors affecting the development of DRE. Second, though we tried to make our assessment of the drug resistance status reliable, it is undeniable that some “DRE patients” may have “pseudo‐resistance”.^49^ Third, the data of several variables were based on physician reports or descriptions of patients or family members; therefore, may lead to possible information bias. Further population‐based prospective studies are needed to clarify the risk factors for DRE among PTE patients fully.

## 5 CONCLUSION

This study found that younger age at the first‐time late PTS onset, focal onset or mixed onset seizure, the presence of SE, and epileptiform discharges during interictal EEG monitoring were risk factors of DRE among PTE patients. A clinical nomogram to predict DRE was developed from the multivariable prediction model, which included these factors. The proposed nomogram achieved significant potential for clinical utility in the prediction of DRE among PTE patients. The risk of DRE for individual PTE patients can be estimated using this nomogram, and identified high‐risk patients might benefit from the comprehensive epilepsy evaluation and the non‐pharmacological therapies at the early stage of PTE.

## AUTHOR CONTRIBUTIONS

All authors contributed to the study conception and design. TTY, XL, LS, RJL were major contributor in the acquisition of data. TTY, JPW, QW analyzed the data. TTY drafted and revised the manuscript and all authors commented on previous versions of the manuscript. All authors read and approved the final manuscript.

## CONFLICT OF INTEREST

None of the authors has any conflict of interest to disclose.

## INFORMED CONSENT

The study was conducted in accordance with the Declaration of Helsinki, and all participants provided informed consent for the use of their medical records.

## Data Availability

The data that support the funding of this study are available from the corresponding author upon reasonable request.
